# Use of medicinal plants by cancer patients at the National Institute of Oncology, Rabat: a cross-sectional survey

**DOI:** 10.11604/pamj.2021.40.18.24992

**Published:** 2021-09-06

**Authors:** Nadia El Orfi, Saber Boutayeb, Bouchra Haddou Rahou, Ahlam Aitouma, Amine Souadka

**Affiliations:** 1 Life and Health Department, University of Medicine and Pharmacy Mohammed V, Rabat, Morocco,; 2 Department of Medical Oncology, National Institute of Oncology, University Mohammed V, Rabat, Morocco,; 3 Research Department, High Institute of Nursing Professions and Technical Health, Rabat, Morocco,; 4 Surgical Oncology Department, National Institute of Oncology, University Mohammed V, Rabat, Morocco

**Keywords:** Cancer, medicinal plants, adverse reactions, reported efficacy

## Abstract

**Introduction:**

the use of medicinal plants has increased significantly in recent years. According to the World Health Organization, 80% of the world's population uses medicinal plants to treat themselves. Our study aims to estimate the prevalence of medicinal plant use by cancer patients, list the different plants and identify their adverse effects cited by users and their reported efficacy.

**Methods:**

this study was realised among 100 patients via a questionnaire with 14-items. Socio-economic and clinical characteristics have been analysed. The bivariate and multivariate analyses have been used to demonstrate the association between the socio-demographic characteristics of the participants, the duration of the disease and the use of medicinal plants.

**Results:**

45% of participants used medicinal plants. The most commonly reported reason for using medicinal plants was cancer cure (22%). During this study, 32 plants were identified. The Honey was the most commonly used (25%), thyme was also consumed at 15%, fenugreek at 13% and garlic at 7%. According to the multivariate analysis, the residence is predictor of medicinal plant use, urban residents used medicinal plants more than rural patients with an OR: 3,098, IC, 95%: [1,183-8,113] and P = 0,021. Fifty patients reported the moderate efficacy of the use of medicinal plants, and 20% described some side effects such as abdominal pain in 34%.

**Conclusion:**

in order to avoid any interaction with oncological drugs and to improve their effectiveness, a great importance must be given to information, education and awareness sessions.

## Introduction

In recent years, medicinal plants (MP) as well as honey as a plant derivative processed by bees [1] and obtained from the combination of the plant and animal world [2] have been accorded great importance as a natural remedy for the treatment of diseases at the international level. According to the World Health Organization, 80% of the world's population uses medicinal plants for their health. In China, herbal preparations represent 30 and 50% and in Germany, 90% of people take a natural remedy [3]. Morocco is known throughout the world for its wealth of aromatic and medicinal plants and its diversity. It has 4200 species of which 800 are endemic and nearly 500 species are used in the medicinal and/or aromatic field [4].

According to a national study, 77.8% of patients consulting a herbalist use MP to treat different diseases [5]. Consumer patients perceive plants as natural and safe products [6], however, some scientific research has shown that plants can contain toxic substances mainly mercury, lead, cadmium, copper, iron, manganese, nickel, zinc and arsenic [7]. Cancer patients are no exception to this rule. At the national level, various studies have shown MP utilization rates in this category of patients and have revealed a rate by 36% of patients [7, 8]. In Turkey, the prevalence of MP use is 68.2% [9]. In China, the utilization rate was 53%. [10]. These natural remedies used may interact with conventional agents and their association with concomitant treatment may have an impact on its efficacy and safety [11]. They can also affect the absorption, distribution, metabolism, excretion and toxicity of drugs [12].

The objective of this study is to estimate the prevalence of MP use in cancer patients followed at the National Institute of Oncology (NIO), and to list the MP used, the information that participants had about the MPs consumed, their adverse reactions and their reported efficacy.

## Methods

**Design and setting of the study:** this is a cross-sectional study conducted at the National Institute of Oncology, particularly in hospitalization services for digestive surgery, gynecomammary surgery, medical oncology, radiotherapy and external services such as outpatient clinics, biological analysis laboratories and day hospitals. This study consists of estimating the prevalence of MP use, listing the different MP used by participants and identifying the adverse effects cited by users and the effectiveness expressed. The duration of this study was one month from March 15^th^ to April 13^th^ 2018.

**Participants:** the sample for the present study used a national average prevalence of 40% calculated from results obtained from national surveys of MP use among cancer patients. The confidence interval was 95% and the margin of error was 10%. The study sample size was 92. A supplement has been made to avoid losses. The study involved both inpatient and outpatient. A total of 100 patients of different sexes, their selection was random. All patients over 18 years of age; undergoing treatment for digestive, gynecologic or other cancers, present during the study period and willing to complete the questionnaire, were included. Exclusion criteria were patients who refused to participate in the study, patients with WHO>3 or hospitalised in palliative care and patients with a pathology that did not allow them to speak.

### Materials

The study was approved by the Ethics Committee for Biomedical Research of the Mohamed V Faculty of Medicine and Pharmacy in Rabat N° 27/18. It is a questionnaire study administered face to face by the investigators participating in the study, it is in French language.

The questionnaire includes socio-demographic and clinical characteristics of patients as well as 14 items relating to the prevalence of MP, their quantity, the reason for use of MP, the person advising this use, the part consumed, their preparation mode, the frequency and duration of use, information on the MP used, their adverse reactions and their effectiveness. It includes closed-ended questions (gender, age, origin, socioeconomic status, occupation, cultural level, type of illness, duration of illness and location of illness) and open-ended questions.

The patients who answered were classified according to their medical coverage into low socio-economic level with medical assistance regime, those with medical coverage insurance and paid patients. For the residence, patients were classified according to an urban environment (from cities) and a rural environment (from rural areas). The disease stage has been classified into metastasic and non-metastasic stages. The plants surveyed were classified according to their type (condiment, food, aromatic plants, medicinal plants and seeds). We have listed honey among the medicinal plants since it is transformed from nectar by bees and therefore it is considered a product derived from plants. The Codex Alimentarius (FAO 2001) as well as Council Directive 2001/110/EC of 20^th^ December 2001 of the European Union define it as a natural sweet substance produced by bees from plant nectar or from secretions from living plant parts or from excretions of foraging insects left on living plant parts.

The duration of the illness was calculated before hospital admission. For age, it has been classified into three bands, from 20 to 40 years old, from 40 to 60 years old and over 60 years old. The variables studied were calculated in terms of frequency and percent.

**Statistical analysis:** the statistical analysis of the data was performed by the SPSS software version 23. Qualitative variables were expressed in frequency. A Pearson’s chi-square test was used to compare MP users and non-users. Univariate analysis was done to determine the association between socio-demographic, clinical parameters and MP use. Multivariate analysis was used to determine factors predictive of MP use. P value <0.05 was used whether the association is statistically significant.

## Results

**Socio-demographic and clinical characteristics:** during our study, 100 patients were surveyed for a 100% response rate. According to the results, women represented 80% of the study population and married people 56%. Patients aged 40 to 60 years were the most represented group (64%). 89% of the participants had no profession. Regarding medical coverage, 77% of the participants were the patients with medical assistance regime, 75% of the patients came from urban areas and 53% of the patients were illiterate. Gynecologic and breast cancers were the most important with 54%. The most commonly reported antecedents were high blood pressure (13%). 40% of the respondents were on chemotherapy treatment at the time of the study. 66% of patients have non-metastatic cancer. Forty-six of the participants had a duration of illness of less than one year (Table 1).

**Table 1:** socio-demographic and clinical characteristics of patients (n=100)

Variables	frequency	Percent
**Age (years old)**		
Less than 40	14	15
40-60	61	64
Over 60	20	21
**Gender**		
Female	77	80
Male	19	20
**Marital status**		
Single	28	28
Married	57	57
Divorced	15	15
**Profession**		
civil servant active	6	6
Retired	5	5
Without	89	89
**Type of social security**		
Patients with medical assistance regime	75	77
Patients with coverage insurance	20	20
Paid	3	3
**Socio-economic level**		
Low level	52	52
Average level	48	48
**Residence**		
Urban	71	71
Rural	29	29
**Educational level**		
Illiterate	49	53
Primary school	14	15
Secondary school	18	20
University	11	12
**Type of cancer**		
Gynecomammary	43	54
Digestive	20	25
ENT: oral cancer, esophagus, ear, neck, nose and thyroid	7	10
Others	9	11
**Medical history**		
High blood pressure	13	41
Diabetes	5	15
Others	14	44
**current treatment**		
Chemotherapy	40	40
Surgery	38	38
Radiotherapy	22	22
**Stage of the disease**		
Non-metastatic stage	66	66
Metastatic stage	34	34
**Duration of the disease**		
less than 1 year	42	46
1 year to 5 years	35	38
More than 5 years old	15	16

**Figure 1:**
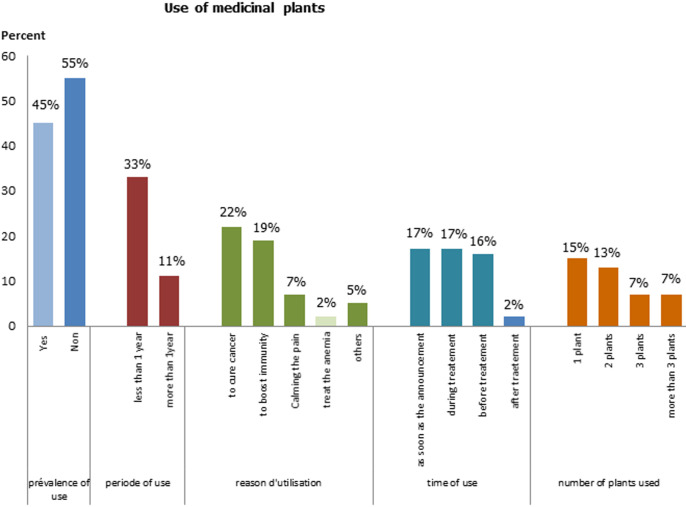
use of medicinal plant; result of the study of use of medicinal plants by cancer patients, Morocco, 2018

**Use of medicinal plants:** of the 100 participants, 45% used medicinal plants. The main reason for use was cancer treatment (22%). The use of MP increases during treatment (36%). Thirty Two percent of patients reported using MP less than one year. The use of a single plant was observed in 36% of the participants (Figure 1).

**Medicinal plants used:** during this study, 32 plants were identified belonging to different categories and showing the richness of the traditional Moroccan pharmacopoeia in cancer patients in the Rabat region and followed up at NIO. The most used of these plants were honey (29%), thyme (18%), fenugreek (15%), nigella seed (8%) and garlic (8%). The most commonly used part was seeds (29%), the amount consumed was an additional spoon at 61%, the way MP was prepared was infused (29%) and mixed with honey at 29%. About 50% of users consumed MP once a day (Table 2).

**Table 2:** type of MP, quantity, part used, preparation method and frequency of use

Plants	Frequency	Percentage
**Type of MP used**		
Honey	25	29
Thyme	15	18
Fenugreek	13	15
Garlic	7	8
nigella seed	7	8
Turmeric	3	4
White marrube (meriwet)	3	4
Mint pouliot (flio)	2	2
Aquilariamalaccensis (aghriss)	2	2
Autres	8	10
**Part used**		
Root	14	23
Sheet of paper	14	23
Seed	18	29
Stem	4	7
Mixing	11	18
**Quantity used**		
Handle	10	23
Spoon	27	61
Glass	7	16
**Preparation method**		
Infusion	17	29
with tea	8	14
Powdered	4	7
with the power supply	12	21
with honey	17	29
**Frequency of use**		
Once a day	22	50
2 times/day	4	9
1 time/week	3	7
2 times/week	10	23
Once a month	4	9
2 times/month	1	2

**Information on PM, person advising on the use and acquisition of MP:** about 58% of the users among the 100 participants had information about the MPs consumed, of which 31% stated that they are useful for strengthening immunity. The use of these natural remedies was mainly requested by the participant's entourage (66%). For the supply of MP, 80% of 100 respondents did not change their supplier (Table 3).

**Table 3:** information on PM, person advising on the use and acquisition of MP

Variables	frequency	Percent
**Information**		
Yes	26	58
No	19	42
Beneficial and effective for health	7	24
Promotes the healing of cancer	4	14
Strengthens immunity	9	31
Calms and soothes pain including gastric pain	2	7
Cleans the neck and purifies the Blood	2	7
Treats anemia, fatigue and Flu	3	10
Other	2	7
**Person advising MPs**		
Colleague	7	16
Yourself	2	5
Physician	1	2
entourage	29	66
internet	4	9
Media	1	2
**Change of supplier**		
Yes	36	80
No	9	20
**Purpose of supplier change**		
search for quality	2	33
Digging in the wilderness	2	33
most convenient	1	17
Product availability	1	17

**Figure 2:**
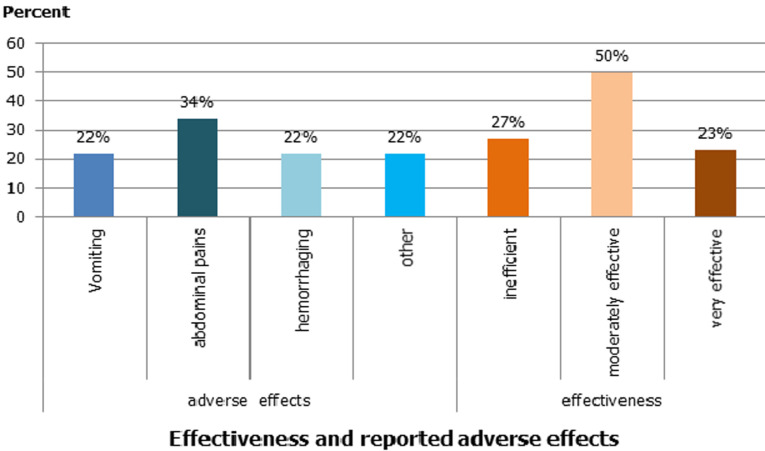
effectiveness and reported adverse effects. Result of the study of use of medicinal plants by cancer patients, Morocco, 2018

**Effectiveness and adverse effects of MP:** certainly, 50% of 100 participants found MPs moderately effective and 20% of participants reported adverse effects, including abdominal pain (34%) (Figure 2).

**Predictors of MP use:** a univariate analysis was performed to analyze the association between socio-demographic and clinical characteristics and MP use. Results showed a very significant association with the residence (P=0.02), participants from the urban environment were the most frequent users of MP. A multivariate analysis showed that the independent factor predicting the use of MP is the residence (OR: 3,098, IC 95%: [1,183-8,113], P=0,021) (Table 4).

**Table 4:** comparison of variables between MP users and non-users

Variable	User (n=45)		Non-user (n=55)		Univariat	Multivariate		
	**n**	**%**	**N**	**%**	**P**	**OR**	**IC**	**P**
**Gender**								
Female	32	71	46	84				
Male	13	29	9	16	0.13			
**The residence**								
Urban	37	82	34	62	0.02	3,098 [1,183-8,113] 0,021		
Rural	8	18	21	38				
**Profession**								
Without	38	84	51	93	0.1			
Active	5	11	1	2				
Retired	2	5	3	5				
**Socio-economic level**								
Low level	28	62	24	44	0.1			
Average level	17	38	31	56				
**Educational level**								
Illiterate	22	49	31	56				
Primary school	8	18	7	13	0.5			
Secondary school	11	24	9	16				
University	4	9	8	15				
**Stage of the disease**								
Non metastatic stage	29	64	37	67	0.8			
Metastatic stage	16	36	18	33				
**Family situation**								
Single	10	22	18	33	0.1			
Married	25	56	32	58				
Divorced	10	22	5	9				
**Medical coverage**								
Patients with medical assistance regim	10	22	10	18	0.8			
Patients with coverage insurance	34	76	43	78				
Paid	1	2	2					
**Diagnosis**								
Gynecological-cancer	23	51	31	4				
Mammary								
Digestive cancer	12	27	13	56				
ENT: oral cancer, esophagus, ear, neck, nose and thyroid	7	15	3	24	0.2			
Others	3	7	8	15				
**Age**								
< than 40 years old*	5	11	10	18	0.6			
40-60 years old	31	69	33	60				
> than 60 years old	9	20	12	22				
**Duration of disease**								
Loss than 1 year	18	40	28	51	0.6			
1-5 years	19	42	18	35				
More than 5 years	8	18	8	14				

## Discussion

Our study revealed the use of MP by patients during conventional treatment. The rate of their use was higher (45%) than that shown in other studies [7]. Despite advice and prescriptions from oncologists reporting a ban on taking MP, the rate of use remains increasing, this can be explained by the recent development of media that encouraged the use of MP to treat chronic diseases as well as the influence of family and friends, this has been confirmed by other studies in different countries [9, 10, 13].

A variety of plants (32 plants) were identified during our study belonging to different classes. Some studies have cited and shown the use of a different package of MP by patients as remedies, supplements and functional foods [7, 14, 15]. In our study honey is the most commonly used product, as are the results of was the most commonly used, similar to the results of a previous study [8]. Thyme, fenugreek and garlic were also consumed by patients; their use has been demonstrated in other similar studies [16-18].

The results of our study showed that MP is used not only in seeds, which was the most consumed part, but also in leaves and roots, which is perfectly in line with another study that showed that leaves, fruits and seeds are the essential ingredient in the preparations used [19].

Our study showed that the environment of origin is a predictive factor in the use of MP by cancer patients. Indeed, participants from urban areas were more likely to use natural remedies to treat themselves, this may be due to the availability and accessibility of MP. Participants reported adverse events (AEs) (20%) related to the use of MP, this rate was somewhat similar to that reported in other previous studies [7, 10]. These AEs can be explained by the lack of knowledge of the MPs regarding the dosage, the preparation method and the part used as well as the random use since the Moroccan market is rich in MP and their sale is not subject to any control or regulation. However, this rate may be low estimated due to the non-medicalised surveillance of the MP intake. The results of our study showed that patients (50%) found MPs moderately effective, as demonstrated in another previous study [15].

**Limits:** the limitations of this study may be related to the under-reporting of patients regarding the use of MP and the low number of participants, and the potential underreporting of side effects due to uncontrolled MP use and non-medicalized surveillance.

## Conclusion

The use of MPs in Morocco, especially in the Rabat region, is very much in demand by cancer patients. Despite the advice provided by oncologists, the use of these remedies for treatment remains high. Our study counted 32 species consumed by these patients. The residence is a predictor of the use of MPs, patients living in urban areas are more likely to consume MPs. Honey, thyme, fenugreek, black cumin seed and garlic are the most commonly used herbs. Most patients believe in the efficacy of MP without considering side effects or interactions with conventional treatment. Pending further studies to justify this efficacy, adverse effects and interactions. It is necessary to be vigilant with patients using these plants during treatment and to adopt strategies to sensitize and educate the patient and his entourage.

### What is known about this topic

- Cancer patients under conventional treatment should not consume herbal medicines;
- Herbal medicines consumed may cause interactions with conventional treatment and result in side effects;
- The medicinal plants consumed can influence the effectiveness of the treatment and delay healing.

### What this study adds

- Patients have reported side effects from herbal medicines, particularly abdominal pain;
- The rate of consuming medicinal plants in study patients is high at 45%;
- 32 plants were identified in our study.

## References

[1] Bonté, Frederic A Rossant, JC Archambault, Desmoulière Alexis (2011). Miels et plantes: De la thérapeutique à la cosmétique. La Phytothérapie Européenne.

[2] Marchenay P (1988). Miels, miellats, miellées. Journ d´agric trad et de Botanique Appl.

[3] OMS 2003 Medicine traditionnelle.

[4] Bachar M, Zidane L, Rochdi (2016). Ethno-medicinal and traditional Phytotherapy of plants used in Bouhachem Natural Regional Park “Rif of Morocco”-case of Tazroute district. J Mater Environ Sci.

[5] Zeggwagh AA, Lahlou Y, Bousliman Y (2013). Survey of Toxicological Aspects of Herbal Medicine Used by a Herbalist in Fes, Morocco. Pan Afr Med J.

[6] Bent S (2008). Herbal Medicine in the United States: Review of Efficacy, Safety, and Regulation. J Gen Intern Med.

[7] Chebat A S, Skalli H, Errihani L, Boulaâmane M, Mokrim T, Mahfoud R, Soulaymani A, Kahouadji (2014). Prevalence Study of Adverse Effects Associated with the Use of Medicinal Plants at the National Institute of Oncology (Morocco).

[8] Tazi I, Nafil H, Mahmal L, Harif M, Khouchani M, Saadi Z (2013). Complementary Medicine in Cancer Patients under Treatment in Marrakech, Morocco: a Prospective Study. Bulletin de la Societe de Pathologie Exotique (1990).

[9] Tuna S, Dizdar O, Calis M (2013). The Prevalence of Usage of Herbal Medicines among Cancer Patients. J BUON.

[10] Liu TG, Xiong SQ, Yan Y, Zhu H, Yi C (2012). Use of Chinese Herb Medicine in Cancer Patients: A Survey in Southwestern China. Research Article, Evid Based Complement Alternat Med.

[11] Stedman C (2002). Herbal Hepatotoxicity. Semin Liver Dis.

[12] Bush MT, Rayburn KK, Holloway SW, Sanchez-Yamamoto DS, Allen BL, Lam T (2007). Adverse interactions between herbal and dietary substances and prescription medications: a clinical survey. Altern Ther Health Med.

[13] Oyunchimeg B, Hwang JH, Ahmed M, Choi S, Han D (2017). Complementary and Alternative Medicine Use among Patients with Cancer in Mongolia: A National Hospital Survey. BMC Complement Altern Med.

[14] Kabbaj FZ, Meddah B, Cherrah Y (2012). Ethnopharmacological Profile of Traditional Plants Used in Morocco by Cancer Patients as Herbal Therapeutics. Phytopharmacology.

[15] Clement YN, Mahase V, Jagroop A, Kissoon K, Maharaj A, Mathura P (2016). Herbal Remedies and Functional Foods Used by Cancer Patients Attending Specialty Oncology Clinics in Trinidad. BMC Complement Altern Med.

[16] Engdal S, Steinsbekk A, Klepp O, Nilsen OG (2008). Herbal Use among Cancer Patients during Palliative or Curative Chemotherapy Treatment in Norway. Supportive Care in Cancer.

[17] Damery S, Gratus C, Grieve R, Warmington S, Jones J, Routledge P (2011). The Use of Herbal Medicines by People with Cancer: a Cross-Sectional Survey. Br J Cancer.

[18] Picking D, Younger N, Mitchell S, Delgoda R (2011). The Prevalence of Herbal Medicine Home Use and Concomitant Use with Pharmaceutical Medicines in Jamaica. J Ethnopharmacol.

[19] Jaradat NA, Al-Ramahi R, Zaid AN, Ayesh OI, Eid AM (2016). Ethnopharmacological Survey of Herbal Remedies Used for Treatment of Various Types of Cancer and Their Methods of Preparations in the West Bank-Palestine. BMC Complement Altern Med.

